# Evaluation of Eating Habits and Quality of Life in Postbariatric Surgery Patients and Their Family Members: A Case-Control Study

**DOI:** 10.1155/2021/6657567

**Published:** 2021-03-02

**Authors:** Carla Ibrahim, Joane Matta, Kàtia Lurbe i Puerto, Yonna Sacre

**Affiliations:** ^1^Holy Spirit University of Kaslik, Faculty of Arts and Sciences, Department of Nutrition and Dietetics, P.O Box 446 Jounieh, Mount Lebanon, Jounieh, Lebanon; ^2^Nutrition & Diabetes Unit, European Hospital Georges Pompidou, Paris, France

## Abstract

**Background:**

Obesity is a major health problem worldwide. Today, bariatric surgery is considered as the last option and most effective treatment for severe obesity (BMI ≥40 kg/m^2^ or BMI ≥35 kg/m^2^ with metabolic conditions).

**Aims:**

We aimed to evaluate the medium-term effect (>36 months) of bariatric surgery and assess postbariatric nutritional and lifestyle management among Lebanese patients who underwent bariatric surgeries in Jbeil and Keserwan hospitals.

**Methods:**

This study is a quantitative case-control study. The case group includes a couple of patients who have undergone sleeve or bypass surgery 6 months ago and above, along with the main family caregivers. The control group constitutes a couple of obese patients with BMI ≥30 kg/m^2^ who did not proceed to any surgical intervention with their main caregivers. The final samples consisted of 53 cases and caregivers and 50 controls and caregivers. The assessment was made by an online questionnaire.

**Results:**

Compared to obese patients, bariatric patients were less likely to have high energy intake (54% versus 34%, *P* value 0.012). Moreover, 35.8% of the caregivers of bariatric patients had a low physical activity level compared to those of the control group who had a lower level (70%). In addition, cases' main caregivers (75.5%) had much higher quality of life compared to the cases (56.6%), and also, higher quality of life was seen among the case's main caregivers (75%) compared to the controls (72%).

**Conclusion:**

In Jbeil and Keserwan regions of Lebanon, there is a lack of postbariatric nutritional and lifestyle management leading to less desirable outcomes in the medium to long term. A set of recommendations are developed based on this study.

## 1. Introduction

According to the World Health Organization (WHO), obesity, defined as body mass index (BMI) ≥30 kg/m^2^, presents a major health problem worldwide. It has been classified as the sixth significant factor contributing to mortality and morbidity from chronic diseases [1]. The Middle East region has reached a higher rate of overweight and obesity—66 to 75% of adults and 25 to 40% of children—and adolescents are estimated to be overweight or obese in Kuwait, Qatar, Saudi Arabia, and Bahrain [2]. Lebanon, this small middle-income country, is experiencing an accelerated rate of nutritional changes which can lead to an increase in obesity levels and other noncommunicable diseases [3]. Indeed, from 1997 to 2009, obesity rates in Lebanon have significantly increased from 17.4 to 28.2% among adults [4].

Lifestyle modification based on behavioral and nutritional changes involving dietary readjustment and an increase in physical activity (PA) is the initial treatment for obesity. But the results of this treatment are often disappointing (95% of the time) with rarely significant weight loss or, in the vast majority of cases, weight regain (the yoyo-effect diet) in the medium and long term [5]. The secondary treatment is pharmacotherapy [6] taken alongside with lifestyle management [7]. Weight regain is also observed whenever the drug therapy is stopped [7].

Today, bariatric surgery (BS) is considered as the last option and the most effective treatment for severe obesity (BMI >40 kg/m^2^ or BMI >35 kg/m^2^ with metabolic conditions) [6]. In Lebanon, sleeve gastrectomy (SG) and gastric bypass (GB) are the most common surgeries used to treat obesity [3]. In fact, this type of treatment is the only way to undergo in morbidly obese patients leading to significant and lasting results of weight loss and improvement of comorbidities [8]. In addition, BS is associated with an improvement in psychological well-being which affects positively the quality of life (QL) of operated patients [9, 10]. Here comes the hypothesis of this study: nutritional status, PA, and QL is better among patients who have undergone BS compared to nonoperated obese people and their caregivers. Also, age, sex, and marital status may explain differences in lifestyle behavior after the surgery. Yet, nutritional management is a key element for good BS preparation and its postoperative success [11]. A reduction of energy intake (EI) and an adaptation of healthy and balanced diet were observed after 6 months of BS. However, these transformations diminish 4 years after surgery [12]. This is related to the lack of nutritional management and follow-ups after BS that leads to weight regain [13]. This can describe the situation in Lebanon.

Concerning the effect of BS on the family members of operated patients, there are no enough data. A few authors who have investigated the effects of BS on the eating behavior of close family members have found that one out of two spouses experienced a change in weight [14]. But lifestyle management (balanced diet and physical activity) can have a positive influence on the behavior of close family members of operated people, who themselves have an increased risk of being obese [13, 15].

Very few are known in Lebanon about the practices, nutritional and behavioral management, being conducted after BS, and the level of maintaining a healthy lifestyle among postsurgery patients after a certain period of time. Hence, the general aim of this study is to evaluate medium- to long-term effects of BS and assess postbariatric nutritional and lifestyle management among Lebanese operated patients in the hospitals of Jbeil and Keserwan. The secondary aim is to compare eating behaviors, QL, and PA after BS in patients and their caregivers with a control group.

This study is inspired by the SociOb Study conducted in France [16].

## 2. Methods

This study was a quantitative case-control study, conducted in the regions of Jbeil and Keserwan. To collect the samples required for the study, the following hospitals were visited: Notre Dame Maritime Hospital, Notre Dame de Secours Hospital, Saint Louis Hospital, Saint Georges Ajaltoun Hospital, Notre Dame du Liban Hospital, and KMC Hospital.

**Figure 1:**
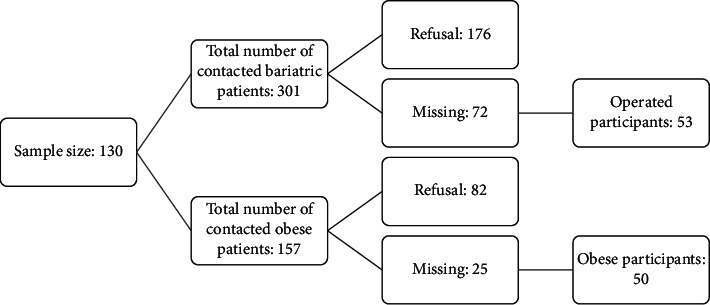
Sampling recruitment.

A total of 103 patient-caregiver dyads were recruited (Figure 1), summing up 206 subjects aged between 23 and 63 years. Among the patients recruited, 53 had undergone BS and 50 were obese participants. Inclusion criteria for the case group (bariatric patients) were as follows: aged 18+, have undergone SG or GB before 6 months and above, and have a main family caregiver willing to participate in the study. For the control group (obese patients), inclusion criteria were as follows: aged 18+, BMI ≥30 kg/m^2^, and a main family caregiver willing to participate in the study. Main caregivers are close family members who live in the same household and share the same lifestyle.

Bariatric patients and obese participants were recruited from the hospital's external/private clinics of dietitians/doctors from July 4 to November 6, 2019.

In order to calculate the sample size of patients who underwent BS, we used the Krejcie and Morgan's formula in Goyette: *n = X2NP(1 − P)/d2(N − 1)+X2P(1 − P)* = 130 participants [17].

An online survey was developed and shared with the participants who consented to participate in the study. This survey comprised five sections: sociodemographic characteristics, anthropometric measurements, food consumption, PA, and QL. The same survey was distributed to the case and control groups and their main caregivers.

To measure the eating habits, the total food consumption per day was calculated, and then, the researcher referred to “MyPlate” and created the score for each food category. For measuring QL and eating behavior, a five Likert scale was used. This questionnaire comprised five categories: physical impact, psychosocial impact, sex life, comfort with food, and diet experience. The IPAQ questionnaire was used to assess the PA status.

The survey was a reduced version of the questionnaire used in the SociOb study [16] and was adapted to the Lebanese population.

The data were analyzed using SPSS software version 21. A paired sample *T*-test was used to compare the body weight and the EI before and after the surgery. To compare eating behavior, QL, and PA before and after the surgery, we used the chi-square test. A regression analysis was conducted to study the correlation between age, sex, marital status, BMI, and QL of the bariatric patient group and their level of PA and EI.

## 3. Results

Table 1 summarizes the sociodemographic and anthropometric characteristics of the studied population. Regarding the case group, 26 patients conducted SG (49.1%) and 27 conducted GB (50.9%) with a mean age of 34.43 years ± 9.30 (SD) and mean BMI of 29.35 ± 6.77 (SD). As for the control group, 40% were female and 60% were male with a mean age of 48.06 years ± 14.30 (SD) and mean BMI of 34.22 kg/m^2^ ± 4.21 (SD).

### 3.1. Food Frequency

The study compares the pattern of EI between the controls and the cases. A statistical difference in the high level of EI was observed (*P* value: 0.012; 95% CI): the cases had a mean EI of 1,769.96 ± 1,403.65 calories per day after surgery compared to the mean EI of 3,710.54 ± 2,470.05 calories per day of the control (*P* value: 0.001). The results showed that 34% (*n* = 18) of patients who had undergone BS had high EI, compared to 54% (*n* = 27) of obese patients. Among 18 patients who had conducted BS, 10 of them had conducted SG and 8 had conducted GB with a mean time of surgery of 3.5 years.

Concerning food frequency (Table 2), 60.4% (*n* = 32) cases had low intake of fruits, yet 58% (*n* = 29) of controls had high intake of fruits (*P* value: 0.001). As for the intake of vegetables, both cases and controls had high consumption of vegetables, and no statistically significant difference was noted. Furthermore, both cases and controls had high consumption of junk food. Although there was a slight difference in percentages of cases and controls highly consuming junk food, this difference was not statistically significant (*P* value: 0.062).

### 3.2. Physical Activity

Regarding the level of PA, no significant difference between the case and control patients' group was found between the bariatric patients and their main caregivers.

In contrast, the difference between the level of PA of the control group and their main caregivers was significant (*P* value: 0.044; 95% CI). 64% (*n* = 32) of the control group had low level of PA, compared to 70% (*n* = 35) of their main caregivers. Moreover, a significant difference resulted due to the comparison of the level of PA between the cases' main caregivers and the controls' main caregivers (*P* value: 0.001; 95% CI): 70% of the control's main caregivers had lower PA compared to 35.8% of cases' main caregivers (Table 3).

### 3.3. Quality of Life

To assess the QL of participants, the following variables were studied: physical impact, psychosocial impact, sex life, comfort with food, and diet experience.

As mainly reported in all the groups, their weight had a low impact on their physical well-being, psychosocial well-being, sex life, comfort with food, and diet experience.

The difference between the impact of the weight on the diet experience of the case group and their main caregivers was found to be significant (*P* value: 0.008). 43.4% of the case group experienced lower impact of their weight on their diet experience compared to 71.7% (*n* = 38) of their main caregivers. Also, there was a significant difference between the impact of weight on the psychosocial wellbeing of the main caregivers of bariatric patients and the main caregivers of obese patients (*P* value: 0.024; 95% CI). 13.2% (*n* = 7) of the main caregivers of the cases reported that their weight had high impact on their psychosocial wellbeing, while none of the main caregivers of the controls had high impact.

Comparing the results of the QL between the cases and their caregivers, another statistical difference is observed (*P* value 0.034: 95% CI). The percentage of main caregivers (75.5%) having high QL is higher than that of the cases (56.6%). Yet the difference in the pattern of QL between the control group and their main caregivers was not statistically significant (*P* value: 0.173). The difference between the percentages of QL of both groups of the main caregivers was statistically significant (*P* value: 0.006). 75% (*n* = 40) of the cases' main caregivers had high QL, while 72% (*n* = 36) of the controls' main caregivers had high QL. This indicated that the QL of the main caregivers of the case group was higher than the QL of the control's main caregivers (Table 4).

### 3.4. Results of the Regression Analysis

Referring to the literature, the outcomes of BS on the short term are remarkably guaranteed. But apparently and based on the results of our study, the medium- and long-term effects are not well maintained because of the lack of postbariatric lifestyle management. Therefore, the focus was on the PA and the EI because these variables are two basic factors to maintain a healthy lifestyle. Hence, a regression analysis was conducted. We accounted for age, sex, BMI, marital status, and QL of the bariatric patients. The results were found to be nonsignificant. Yet, it is important to go through the main findings as follows.

#### 3.4.1. Physical Activity

Based on the results of the conducted regression analysis among bariatric patients (Table 5), females are 0.507 times less likely to do PA than males. Also, the married are 0.453 times less likely to do PA than single patients. Yet, the separated/divorced patients were 1.127 times more likely than single cases to conduct PA. However, since the odds ratio is nonsignificant, we should not conclude a substantial relationship of the two variables. As for the age and the BMI, for one unit of increase in the age and the BMI, the odds of cases, doing PA increase by 1.047 and 1.030 times, respectively. In addition, patients with high EI were 1.065 times more likely than the ones with low EI to conduct PA.

#### 3.4.2. Energy Intake

Based on the results of the conducted regression analysis among bariatric patients (Table 5), the females were 1.491 times more likely to have high EI than males. Also, the married were 1.994 times more likely to have higher EI than single patients. As for the age and the BMI, for one unit of increase in the age and the BMI, the odds of having higher EI increased by 1.059 and 1.000 times, respectively. For the QL and EI, patients who reported having moderate QL were 3.518 times more likely than the ones with low QL to have high EI. Similarly, patients who reported having high QL were 3.696 times more likely than the ones with low QL to have high EI. In addition, patients with moderate PA were 0.739 times less likely than the ones with low PA to have high EI.

## 4. Discussion

The hypothesis of this study mentioned that nutritional status, PA, and QL is better among patients who have undergone BS. Yet, the results of our study revealed that the long duration of the intervention was considered to affect negatively the results of the surgery.

As mentioned earlier, one-third of the bariatric patients goes on having high EI, which was considered relatively to be a high number and set a question mark on the impact of the surgery on the eating habits of patients. This was related to the lack of nutritional management and follow-ups after BS that leads to shortage in dietary changes and caused an increase in calorie intake and induced weight-regain [18]. BS appears to slow dietary compulsions regarding sugary and fast foods [19]. Yet, operated patients tended to consume high intake of junk food after one year of the surgery. Besides, a high EI is observed in obese patients who have not undergone BS, which can justify the persistent habits that reoccur after the surgery. Thus, returning to the previous eating habits and not changing the lifestyle will mislead the outcome of BS [20]. Furthermore, half of the main caregivers of obese patients had high EI, while more than half of the main caregivers of operated patients had low EI. Based on the literature, obesity is contagious. Obese people can affect their family members and lead them to have higher calorie consumption. This goes reversely the same with people living with bariatric patients who can affect their family members and induce them to balance their diet, causing a positive effect on the weight [13, 21].

Moreover, more than one-third of main caregivers of bariatric patients had low PA. In line with Janik et al.'s [10] study, since lifestyle management can influence positively the close family members of operated people, a low PA level of main caregivers could reflect a low PA of the cases. Therefore, on a short-term basis, BS is effective, but sedentary lifestyle and inactivity can lead to serious failure in the long-term basis [22]. Gradaschi et al. [23] demonstrated that a consistent exercise can affect positively the weight loss status. Yet, a decrease in PA levels was observed on the long-term evolution (ten years) of weight and weight gain [24]. This could explain in this study why the participants showed less desirable outcomes of the surgery in the long term.

Furthermore, regarding the QL, Woodard et al.'s study [15] showed that, for beneficial outcomes to occur, weight loss after surgery must be closely monitored and depend on many factors including changes in eating behaviors, food frequency, and PA. An altering QL is related to weight gain due to low PA and recurrence of preoperative eating habits [25, 26]. In our study, less than half of bariatric patients experienced lower impact of their weight on their diet experience compared to approximately three quarters of their main caregivers. Additionally, a high QL of bariatric patients was reported, yet three quarters of their main caregivers showed higher QL. This is related to the positive influence of lifestyle management on the close family members of operated people [10]. Consequently, the cases' caregivers may have improved awareness on the benefit of healthy eating practices. Besides, a higher QL of the case's main caregivers was stated compared to the controls' caregivers. These results can be explained as follows: a better QL is reflected in patients who underwent BS compared with nonoperated obese patients [10]. Another interesting finding was reported in our study: more than one-tenth of the cases' main caregivers reported that their weight had a high impact on their psychosocial wellbeing. This may be related to a greater psychological pressure on the main caregivers in order to maintain their weight when their close relatives underwent the operation.

Although the results of the regression analysis were found to be nonsignificant, they provided few insights concerning some characteristics of the studied groups. Age could be a confounding factor and can affect the results of BS. In addition, after 3.5 years of the surgery, operated patients with high EI (high intake of junk food) were more likely to conduct PA than patients with low EI. Referring to the literature, active people had higher EI than inactive people [27]. This can be related to the increase of the appetite hormones after exercise in some individuals [27]. Therefore, people who underwent BS should conduct a PA simultaneously with a balanced diet in order to improve their weight loss. A high calorie intake is related to a poor nutrition which affects negatively the QL [28]. In our study, patients having higher calorie intake are more prone to have moderate QL. Hence, the regression analysis put in evidence that not only age, sex, marital status, and QL were accounted for behavioral changes but also these changes depended also on environmental issues (major issue in Lebanon), defined by an alteration in the organization of healthcare services that leaded to the lack of follow-ups.

## 5. Study Limitations

The main limitations are as follows: time, limitation of resources, and limited number of samples. This study was a pilot study that might pave the gap for other national studies about this topic. This study is a case-control study, which lacks long-term follow-ups for the cases.

## 6. Conclusion

Obesity is a health problem that might affect the health of people in various ways. Not all people can avoid being obese and find themselves stuck in this state. Some people are left with one last solution, which is to conduct BS. Yet to obtain the most sustainable results from the surgery, patients should be supplied with a package of care provided by a multidisciplinary task force. Various factors affect the provision of such comprehensive package of care for patients. Based on the findings of this study, we conclude that, in Jbeil and Keserwan regions of Lebanon, there is a lack of postbariatric nutritional and lifestyle management leading to less desirable outcomes on the medium and long term. Despite conducting BS, sedentary patients and patients who do not change their lifestyle were not able to maintain the results of their surgery and some of them refollowed nonhealthy eating habits. Therefore, proper and timely follow-up is crucial and a key element for better results and longer lives after BS. The benefits of this surgery exceeded the risks for most of the patients. Yet the surgery is not a miracle, it is just a tool that helps and promotes weight loss, and patients should work on their own to change their lifestyle and eating habits to reach long-lasting outcomes.

### 6.1. Recommendations

A group of multidisciplinary experts must be integrated in the management of operated patients. However, a better involvement of the dietitian in the multidisciplinary management of BS should be taken into consideration and the proposal of a dietetic care strategy should be adjusted to each step of the surgery. Thus, the following recommendations are presented:

- Dietary management must be designed in 3 stages (preoperative, perioperative, and postoperative) to maintain or restore good nutritional, informative, and educational status and to avoid postoperative complications.
- Follow-up consultations during the first year must be scheduled at 3, 6, and 12 months and then depending on the particular situation of the patient. This participates in weight loss and its maintenance and also in the good nutritional status of patients by maintaining a balanced diet and sufficient intake of macro- and micronutrients.
- Integrate family members in the nutritional management of operated patients: by preparing the food together, eating together, and sharing the same lifestyle. This affects positively the results of the surgery.
- Conducting a longitudinal study that targets all the Lebanese regions and studies the lifestyle of the same patient before and after the surgery is important to carry relevant nutritional and dietetic management.

## Figures and Tables

**Table 1:** Sample characteristics—sociodemographic and anthropometric information.

Variable	Population							
	Case (patients) (*N* = 53)		Case (main caregivers) (*N* = 53)		Control (patients) (*N* = 50)		Control (main caregivers) (*N* = 50)	
	Number	Percentage	Number	Percentage	Number	Percentage	Number	Percentage
*Sociodemographics*								
Age								
Mean age ± SD (Years)	34.43 ± 9.30		43.86 ± 14.77		37.34 ± 14.10		48.06 ± 14.30	
*Sex*								
Male	17	32.1%	16	30.2%	30	60.0%	15	30.0%
Female	36	67.9%	37	69.8%	20	40.0%	35	70.0%
*Marital status*								
Single	27	50.9%	13	24.5%	27	54.0%	10	20.0%
Married	24	45.3%	37	69.8%	22	44.0%	39	78.0%
Separated/divorced	2	3.8%	0	0.0%	0	0.0%	0	0.0%
Widowed	0	0.0%	3	5.7%	1	2.0%	1	2.0%
*Type of surgery*			—	—	—	—	—	—
Sleeve	26	49.1%	—	—	—	—	—	—
Bypass	27	50.9%	—	—	—	—	—	—
*Year of surgery*								
less than 1 year	6	11.3%	—	—	—	—	—	—
1–3 years	32	60.4%	—	—	—	—	—	—
4 years and above	15	28.3%	—	—	—	—	—	—
*Categories of caregivers*								
Mother	18	34%	—	—	—	—	—	—
Spouse: husband	13	24.5%	—	—	—	—	—	—
Sister	12	22.6%	—	—	—	—	—	—
Spouse: wife	5	9.4%						
Son	2	3.8%	—	—	—	—	—	—
Daughter	2	3.8%	—	—	—	—	—	—
Father	1	1.9%	—	—	—	—	—	—
	Mean ± SD		Mean ± SD		Mean ± SD		Mean ± SD	
*Anthropometric measures*								
Weight (kilograms)	83.45 ± 19.55		78.88 ± 18.99		101.95 ± 18.50		79.38 ± 14.20	
Height (meters)	1.69 ± 0.10		1.69 ± 0.12		1.72 ± 0.10		1.65 ± 0.07	
BMI (Kg/m^2^)	29.35 ± 6.77		27.57 ± 5.18		34.22 ± 4.21		29.06 ± 4.68	

**Table 2:** Food frequency—case (patients) vs. control (patients).

Variable	Population				*P* value
	Case (patients) (*N* = 53)		Control (patients) (*N* = 50)		
	Number	Percentage	Number	Percentage (%)	
*Fruits*					
Low	32	60.4	12	24.0	0.001^
Moderate	6	11.3	9	18.0	
High	15	28.3	29	58.0	

*Vegetables*					
Low	19	35.8	12	24.0	0.418
Moderate	4	7.5	4	8.0	
High	30	56.7	34	68.0	

*Junk/processed/unhealthy food*					
Low	4	7.5	1	2.0	0.062
Moderate	6	11.3	1	2.0	
High	43	81.2	48	96.0	

*Energy intake*					
Low	14	26.4	16	32.0	0.012^
Moderate	21	39.6	7	14.0	
High	18	34.0	27	54.0	

Mean energy intake ± SD (Kcal)	1769.96 ± 1403.65		3710.54 ± 2470.05		<0.001^

^^^ *P* values reported in red are significant at 0.05.

**Table 3:** Physical activity.

Variable	Case (patients) (*N* = 53)		Control (patients) (*N* = 50)		*P* value
	Number	Percentage	Number	Percentage

*Physical activity*					0.404
Low	27	50.9%	32	64.0%	
Moderate	15	28.3%	10	20.0%	
High	11	20.8%	8	16.0%	
	Case (patients) (*N* = 53)		Case (main caregivers) (*N* = 53)	
	Number	Percentage	Number	Percentage

*Physical activity*					0.211
Low	27	50.9%	19	35.8%	
Moderate	15	28.3%	16	30.2%	
High	11	20.8%	18	34.0%	
	Control (patients) (*N* = 50)		Control (main caregivers) (*N* = 50)	
	Number	Percentage	Number	Percentage

*Physical activity*					0.044^
Low	32	64.0%	35	70.0%	
Moderate	10	20.0%	14	28.0%	
High	8	16.0%	1	2.0%	
	Case (main caregivers) (*N* = 53)		Control (main caregivers) (*N* = 50)	
	Number	Percentage	Number	Percentage

*Physical activity*					<0.001^
Low	19	35.8%	35	70.0%	
Moderate	16	30.2%	14	28.0%	
High	18	34.0%	1	2.0%	

^^^ *P* values reported in red are significant at 0.05.

**Table 4:** Quality of life.

Variable	Case (patients) (*N* = 53)		Case (main caregivers) (*N* = 53)		*P* value
	Number	Percentage	Number	Percentage

Quality of life					0.008 ^
*Diet experience*					
Low	23	43.4%	38	71.7%	
Moderate	15	28.3%	5	9.4%	
High	15	28.3%	10	18.9%	

	Case (main caregivers) (*N* = 53)		Control (main caregivers) (*N* = 50)	
	Number	Percentage	Number	Percentage

*Psychosocial impact*					0.008 ^
Low	43	81.1%	45	90.0%	
Moderate	3	5.7%	5	10.0%	
High	7	13.2%	0	0.0%	

	Case (patients) (*N* = 53)		Case (main caregivers) (*N* = 53)	
	Number	Percentage	Number	Percentage

*Quality of life*					0.034 ^
Low	6	11.3%	7	13.2%	
Moderate	17	32.1%	6	11.3%	
High	30	56.6%	40	75.5%	

	Case (main caregivers) (*N* = 53)		Control (main caregivers) (*N* = 50)	
	Number	Percentage	Number	Percentage

*Quality of life*					0.006 ^
Low	7	13.2%	0	0.0%	
Moderate	6	11.3%	14	28.0%	
High	40	75.5%	36	72.0%	

^^^ *P* values reported in red are significant at 0.05.

**Table 5:** Physical activity/energy intake—bariatric patients.

Variable	Population: case (patients) (*N* = 53)—physical activity			Population: case (patients) (*N* = 53)—energy intake		
	Adjusted OR	95% CI	*P* value	Adjusted OR	95% CI	*P* value
*Marital status*						
Single (ref)	—	—	0.550	—	—	0.698
Married	0.453	[0.104–1.976]	0.292	1.994	[0.405–9.825]	0.396
Separated/divorced	1.127	[0.053–23.911]	0.939	0.000	[—]	0.999

*Sex*						
Male (ref)	—	—	—	—	—	—
Female	0.507	[0.135–1.900]	0.314	1.491	[0.304–7.316]	0.623

*Age*						
Age	1.047	[0.966–1.134]	0.264	1.059	[0.973–1.152]	0.183

*BMI*						
BMI	1.030	[0.939–1.129]	0.535	1.000	[0.906–1.104]	0.998

*Quality of life*						
Low (ref)	—	—	0.862	—	—	0.580
Moderate	0.900	[0.122–6.644]	0.918	3.518	[0.254–48.677]	0.348
High	0.658	[0.097–4.466]	0.668	3.696	[0.304–44.970]	0.305

*Energy intake*						
Low (ref)	—	—	0.950			
Moderate	0.855	[0.192–3.820]	0.838			
High	1.065	[0.207–5.475]	0.939			

*Physical activity*						
Low (ref)				—	—	0.382
Moderate				0.739	[0.150–3.641]	0.710
High				2.752	[0.480–15.775]	0.256

## Data Availability

The data used to support the findings of this study are available from the corresponding author upon request.
